# *Astragaloside* VI Ameliorates Post-Stroke Depression via Upregulating the NRG-1-Mediated MEK/ERK Pathway

**DOI:** 10.3390/ph15121551

**Published:** 2022-12-13

**Authors:** Xi Chen, Jiangang Shen, Qing Zhou, Xinchun Jin, Haosheng Liu, Ran Gao

**Affiliations:** 1 Department of Core Facility, The People’s Hospital of Bao-an, Shenzhen 518000, China; 2 The Second Affiliated Hospital of Shenzhen University, Shenzhen 518000, China; 3 School of Chinese Medicine, Li Ka Shing Faculty of Medicine, The University of Hong Kong, 10 Sassoon Road, Pokfulam, Hong Kong SAR 999077, China; 4 Department of Human Anatomy, School of Basic Medical Sciences, Capital Medical University, Beijing 100069, China

**Keywords:** post-stroke depression, *Astragaloside* VI (AsVI), NRG-1, MEK/ERK pathway, antidepressant

## Abstract

Background: Post-stroke depression (PSD) has been identified as one of the most commonly occurring complications attributed to stroke. *Astragaloside* VI (AsVI), which is an active Radix Astragali (AR)-derived compound, has been reported to be a potential drug for post-stroke therapy, but its effects on PSD and the underlying mechanisms remain uncovered. Methods: In this study, healthy male SD rats underwent a middle cerebral artery occlusion (MCAO) stroke model. To create a PSD model, these rats were then kept in isolated houses and subjected to chronic unpredictable mild stress. The rats were examined every five days for a series of behavioral tests of depression. The antidepressant properties of AsVI were also investigated in vitro in a corticosterone (CORT)-induced major depression model using a CCK-8 assay. The release of neurotransmitters dopamine (DA)/5-hydroxytryptamine (5-HT) was measured using HPLC. The expression of the neurotrophic factor Neuregulin 1 (NRG-1) in rat brain tissues was detected by immunostaining. The protein expression of NRG-1, p-MEK1, and p-ERK1/2 was analyzed utilizing western blotting. Results: AsVI treatment significantly reduced depression-like behaviors in PSD rats and attenuated the CORT-induced apoptotic cell death in neuronal PC-12 cells. Besides, AsVI treatment remarkably prevented the decrease of the levels of DA and 5-HT in the PSD rat brains and in CORT-induced PC-12 cells. Furthermore, AsVI treatment upregulated the NRG-1-mediated MEK/ERK pathway, which is associated with the improvement of PSD. Conclusions: These findings suggest that AsVI could improve PSD at least partially by upregulating NRG-1-mediated MEK/ERK pathway. AsVI could be a novel therapeutic option for treating PSD.

## 1. Introduction

With a 30–40% mortality rate, post-stroke depression (PSD) is one of the most common neuropsychiatric disorders in stroke patients, characterized by re-occurring long-term low mood. It negatively affects rehabilitation therapy and has caused a substantial economic burden worldwide [1,2,3]. Though significant progress has been made in diagnostic and nursing techniques for PSD, promising treatment still remains limited. Thus, developing drug therapy for PSD is critically important.

Multiple variables, such as social, psychological, and biological social factors, contribute to the development of PSD. The molecular pathogenesis of PSD involves neuroinflammation [4], dysregulation of neurotrophins [5], monoamines [6], and neurogenesis [7]. Neuronal cell loss in the ischemic lesion is also closely associated with the occurrence of PSD [8]. Among these, neurotrophins are essential not only for neuronal survival but also for the development of neural plasticity in the CNS [9]. They are also widely implicated in psychiatric diseases [10]. In PSD models, stimulation of the neurotrophic pathway could lead to an antidepressant effect and functional recovery [11]. Hence, neurotrophins are potential targets for PSD treatment.

Neurotrophic factor Neuregulin 1 (NRG-1) has been known as one of the active elements of the epidermal growth factor (EGF)-like family expressed mainly in glutamatergic neurons as well as in interneurons and astrocytes [12]. NRG-1 performs a vital function in promoting the growth and development of the central nervous system (CNS) [13]^,^ and it modulates the glial and immune responses and promotes neuroprotection and remyelination against central nervous system injury [14,15]. Notably, NRG-1 delays the ischemic cortical damage in transient middle cerebral artery occlusion (tMCAO) [16,17,18,19]. The reduction of endogenous NRG-1 exacerbates neuronal injury in vivo [20]. Depression is intrinsic to psychosis, The prevalence of depressive disorders in schizophrenia has been reported to be around 40%, and they showed some same pathological mechanisms [21,22,23]. NRG-1 levels were found to be lower in the prefrontal cortex of patients with schizophrenia, indicating its potential involvement in depression [24].

During PSD, axons containing 5-HT, NE, and DA between the cerebral cortex and brainstem might be impaired, resulting in neurotransmitter production imbalances throughout the brain [25,26]. Increased DA can induce dopaminergic activation to promote synaptic plasticity and reduce cognitive impairment [27]. DA neurons highly express NRG-1 receptors throughout development into adulthood. NRG-1 would also have an effect on DA neurotransmission by raising extracellular DA levels [28].

In the postmortem frontal cortex and hippocampus of depression and suicide patients, the levels of various effectors of the Ras/ERK (MAPK) pathway were found to be decreased [29,30]. Similar to the findings of postmortem research reports, animal studies showed that suppressing ERK signaling in the prefrontal cortex and hippocampus was enough to elicit depression-like behaviors [31], while the activation of ERK signaling in the hippocampus was required to generate the antidepressant-like impacts of intrahippocampal BDNF infusion [32]. Interestingly, NRG-1 could activate the Ras/MAPK/ERK1/2 pathway to initiate the development and myelination of Schwann cells [33,34]. Therefore, targeting NRG-1-mediated MEK/ERK pathway could be a potential strategy for improving PSD.

Radix Astragali (AR) is a traditional Chinese Medicine herb used for stroke treatment. Compounds extracted from AR show neuroprotective impacts against cerebral ischemia injury [35]. Among these active compounds, astragalosides are a cohort of triterpene glycosides, including AstragalosideI (AsI), Astragaloside II (AsII) and Astragaloside VI (AsVI) types, which could promote axonal regeneration and the reconstruction of neuronal synapses [36]. As the major metabolite of astragalosides, AsVI promotes the skin cells’ proliferation and migration by activating EGFR/ERK signaling pathway in vitro and accelerating the cure of sterile and infected wounds in vivo [37]. Our previous study shows that AsVI activates EGFR/MAPK signaling cascades, promotes the proliferation as well as neurogenesis of neural stem cells (NSCs) in transient cerebral ischemic brains, and improves neurological function repair in rats with post-ischemic stroke [38]. However, it remains unclear whether AsVI could protect against PSD. Our previous chip data suggest that the expression of NRG-1 was down-regulated in the MCAO model cohort and was significantly upregulated by AsVI treatment. These data point to the potential that AsVI may protect against PSD through the modulation of NRG-1.

To test this hypothesis, we constructed the in vivo PSD rat model by stimulating the middle cerebral artery occlusion (MCAO) stroke model ensured by chronic unpredictable mild stress (CUMS) and established the in vitro major depression cellular model by incubating PC-12 cells with corticosterone (CORT). Subsequently, we tested the effects of AsVI on activating the NRG-1-mediated MEK/ERK pathway and improving PSD.

## 2. Results

### 2.1. AsVI Improved the Depressive Behavior in PSD Rats

**Figure 1:**
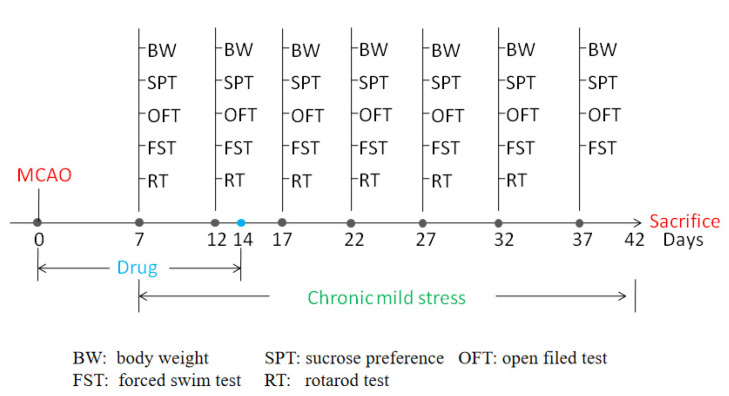
Time schedule of experimental procedures.

**Figure 2:**
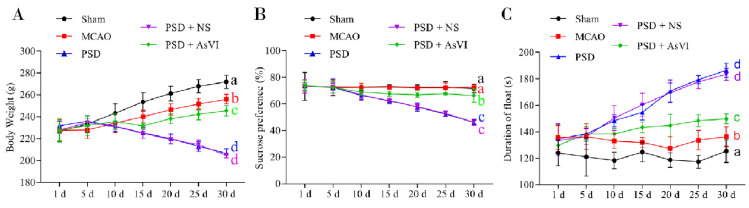
Effects of AsVI on immobility, sucrose preference, and body-weight loss in PSD rat model. (**A**) Dynamical fluctuations in the body weight of the rats. The body weights of rats in the PSD and PSD + NS cohort continually deceased. The body weights of rats in the sham and MCAO cohorts continually increased, but the rat body weights in the sham cohort were heavier than the MCAO cohort. The body weights of rats in the PSD + AsVI cohort decreased prior to the 15th day and afterward increased. The body weights of rats in the PSD + AsVI cohort were lighter as opposed to the MCAO and sham cohort and heavier than PSD and PSD + NS cohorts. (**B**) Dynamical alterations in sucrose preference. The ingestion of sucrose water was reduced in the PSD, PSD + NS and PSD + AsVI cohorts within the initial 10 days as opposed to the MCAO and sham cohorts. Consequently, the ingestion in the PSD + AsVI cohort was found to increase marginally beginning from the 15th day and stayed constant in the days that followed. The ingestion of sucrose water in the PSD cohort was constantly reduced throughout the 30 days. (**C**) Dynamical alterations of immobility. The float time was increased in the PSD and PSD + NS cohorts within the initial 15 days as opposed to the MCAO and sham cohorts. Then, the float time in the PSD + AsVI cohort stayed constant thereafter, and the float time in the PSD + AsVI cohort decreased compared with PSD and PSD + NS cohorts. Data are articulated as Mean ± SD. *n* = 4–6 rats in the cohorts. Distinct letters (a, b, c, d) denote a significant difference in various cohorts, *p* < 0.05.

**Figure 3:**
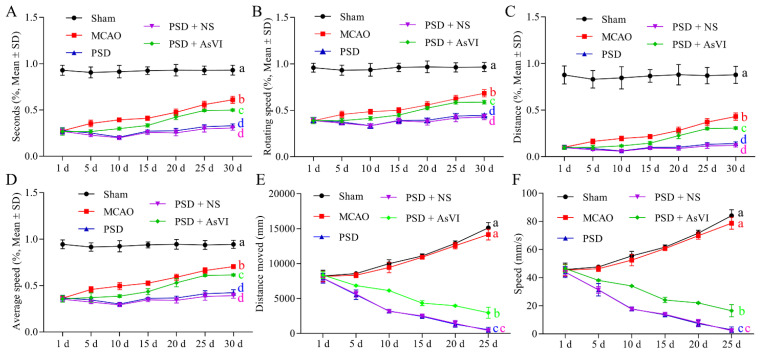
Effects of AsVI on motor functions in locomotor activity and Rotarod test in the open-field experiment in PSD rat model. (**A**) The statistical analysis of the period in the device for each cohort throughout motor detection. (**B**) The statistical analysis of the rotating velocity in each cohort in the course of motor detection. (**C**) The statistical analysis on the distance in each cohort in the course of motor detection. (**D**) The statistical analysis on the average velocity in each cohort in the course of motor detection. (**E**) The statistical analysis on the distance moved in each cohort in the course of the open field test. (**F**) The statistical analysis of the speed in each cohort in the course of the open field test. Data are articulated as means ± SDs. *n* = 4–6 rats in the cohorts. Distinct letters (a, b, c, d) denote significant differences in various cohorts. *p* < 0.05.

To test the effects of AsVI on PSD in vivo, we performed the experiments with SD rats subjected to 90 min transient MCAO ischemia model followed by chronic unexpected mild stress to develop PSD (Figure 1). As expected, PSD rats showed behaviors resembling depression, such as the loss of body weight (BW; Figure 2A), reduced sucrose preference (Figure 2B), immobility (Figure 2C), impaired motor functions (Figure 3A–D), and locomotor activities (Figure 3E,F). After receiving AsVI treatment (2 μg/kg wt), the rats had significantly increased body weight (since the 20th day) and sucrose preference (SPT; since the 15th day), improved immobility in forced swim test (FST; since the 10th day), promoted motor functions in Rotarod test (RT), since the day 10th), and locomotor activities in open filed test (OFT; since the 10th day). These results indicate that AsVI treatment could alleviate behaviors resembling depression in the PSD rat model.

### 2.2. AsVI Increased the Levels of Neurotransmitters in PSD Rats and In Vitro

**Figure 4:**
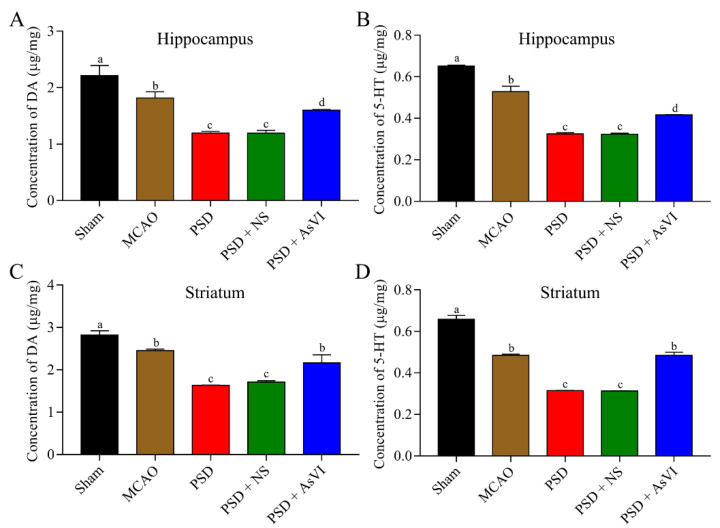
Impacts of AsVI on the brain neurotransmitters in PSD rats. (**A**,**B**) HPLC analysis was carried out to measure the 5-HT and DA levels in the hippocampus from different cohorts. (**C**,**D**) HPLC analysis was carried out to measure the 5-HT and DA levels in the striatum from different cohorts. Data are articulated as Mean ± SD. *n* = 4–6 rats in the cohorts. Distinct letters (a, b, c, d) denote significant differences in various cohorts *p* < 0.05.

Then we further measured the impacts of AsVI on the brain neurotransmitters in PSD rats by using HPLC analysis. As shown in Figure 4A,B, a substantial reduction in the 5-HT and DA levels can be observed in the hippocampus of PSD rats (DA: 1.196 ± 0.029 μg/mg, 5-HT: 0.325 ± 0.006 μg/mg), as compared to the rats in the MCAO (DA: 2.212 ± 0.182 μg/mg, 5-HT: 0.651 ± 0.005 μg/mg) or sham cohorts (DA: 1.816 ± 0.112 μg/mg, 5-HT: 0.529 ± 0.026 μg/mg). Notably, AsVI treatment remarkably reversed the reduction of 5-HT and DA in the PSD rats’ hippocampus (DA: 1.599 ± 0.013 μg/mg, 5-HT: 0.412 ± 0.001 μg/mg). Similar results were also observed in the striatum of PSD rats (i.e., DA in Sham, MCAO, PSD, PSD + NS, PSD + AsVI were 2.821 ± 0.099 μg/mg, 2.450 ± 0.040 μg/mg, 1.633 ± 0.008 μg/mg, 1.712 ± 0.030 μg/mg, 2.165 ± 0.188 μg/mg, respectively; 5-HT in Sham, MCAO, PSD, PSD + NS, PSD + AsVI were 0.658 ± 0.019 μg/mg, 0.484 ± 0.007 μg/mg, 0.315 ± 0.0005 μg/mg, 0.313 ± 0.001 μg/mg, 0.484 ± 0.016 μg/mg, respectively; Figure 4C,D). These results suggest that AsVI could improve PSD rats at least partially by inducing the expression of DA and 5-HT. 

### 2.3. AsVI Regulated the Expression Levels of NRG-1, p-MEK1, and p-ERK1/2 in PSD Rat Brains

**Figure 5:**
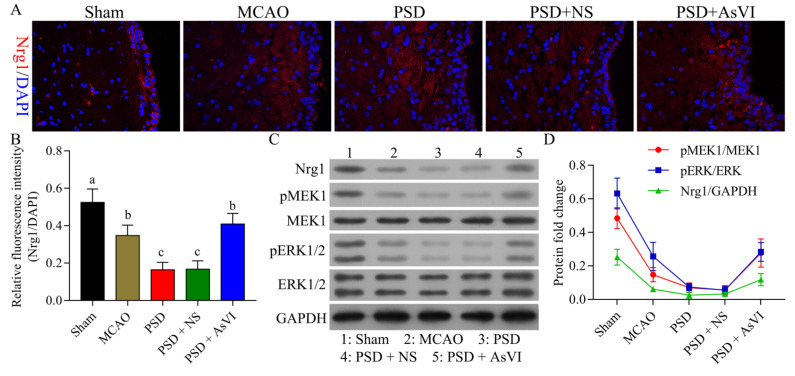
Impacts of AsVI on NRG-1, MEK1, and ERK1/2 expression in the PSD rats’ brain tissues. (**A**) Immunofluorescence was utilized to analyze the expression of neuregulin-1 (NRG-1) in brain tissues from rats in different cohorts. 400× magnification. (**B**) Statistical analysis of the fluorescence intensity of NRG-1 in respective cohorts. (**C**,**D**) The NRG-1, MEK1, p-MEK1, ERK1/2, and p-ERK1/2 expression in the brain tissue of PSD rats was measured utilizing western blotting. GAPDH was employed as an internal control. Data are articulated as means ± SDs. *n* = 3–5 rats in the cohorts. Distinct letters (a, b, c, d) denote significant differences in various cohorts *p* < 0.05.

We then measured the levels of NRG-1, MEK1, and ERK1/2, which are associated with the survival of neuronal cells. As illustrated in Figure 5A,B, the NRG-1 expression was substantially reduced in the brain tissues of PSD rats contrasted with the MCAO cohort. The reduction of NRG-1 was restored with AsVI treatment in PSD rats. In addition, the changes of p-MEK1 and p-ERK1/2 were consistent with the NRG-1 as revealed by Western blot analysis (Figure 5C,D). Such evidence suggests that AsVI regulates the NRG-1/MEK-ERK1/2 signaling pathway, which is associated with improving PSD.

### 2.4. AsVI Improved the CORT-Induced Cellular Model of Major Depression by Upregulating NRG-1-Mediated MEK/ERK Pathway

**Figure 6:**
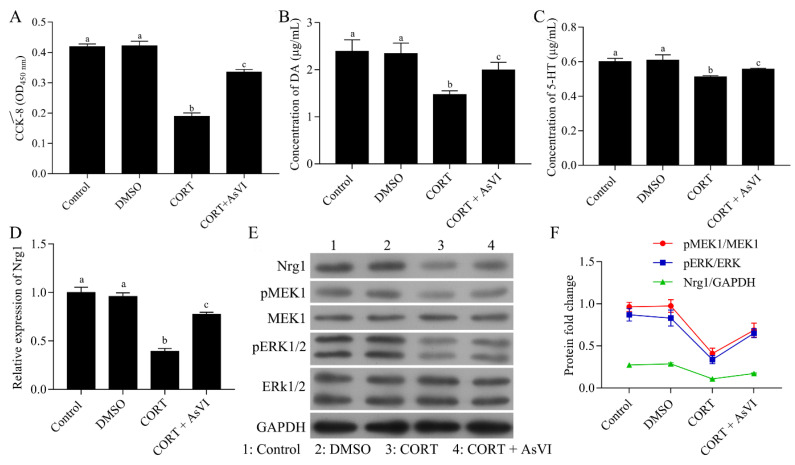
Protective effects of AsVI on CORT-induced PC12 cells. (**A**) The cell viability was measured by a CCK-8 assay. (**B**) HPLC analysis was carried out to measure the DA release levels for various therapies in PC12 cells. (**C**) HPLC analysis was carried out to measure 5-HT release levels for various therapies in PC12 cells. (**D**) A quantitative real-time PCR method was conducted to detect the expression level of NRG-1 mRNA in different treated PC12 cells. (**E**,**F**) Western blot for NRG-1, MEK1, p-MEK1, ERK1/2 and p-ERK1/2 protein for different treatments in PC12 cells. All the experiments were conducted thrice. Data are articulated as Mean ± SD. Distinct letters (a, b, c, d)denote significant differences in various cohorts *p* < 0.05.

Subsequently, we study the effects of AsVI against depression in vitro. We treated PC12 neuronal cells with CORT to construct the major depression model in vitro. CCK-8 assay indicated that AsVI had a significant protective effect on PC12 cells against CORT-induced cytotoxicity (Figure 6A). AsVI increased the DA levels (Figure 6B) and 5-HT levels (Figure 6C) in PC12 cells. Consistent with the in vivo results, the NRG-1, p-MEK1, and p-ERK1/2 expression was considerably down-regulated in CORT-induced PC12 cells but upregulated by AsVI treatment (Figure 6D–F). Our results reveal that AsVI protects PC12 cells against CORT-induced injury, which is associated with upregulating the NRG-1/MEK-ERK1/2 signaling pathway.

**Figure 7:**
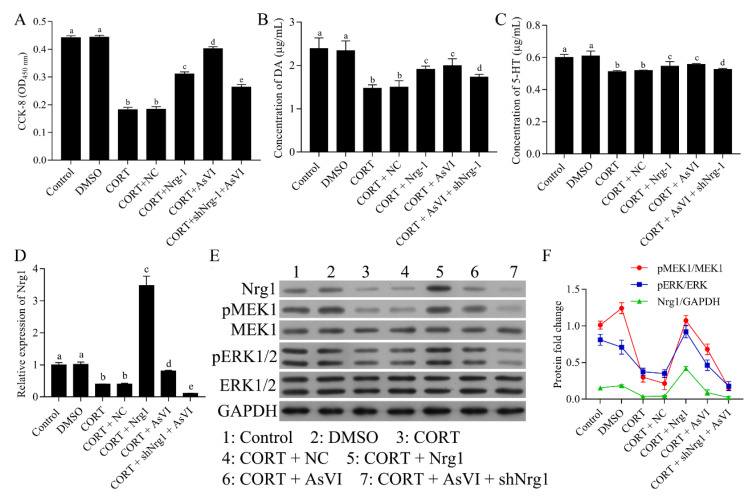
AsVI improved the CORT-induced cellular model of major depression by upregulating the NRG-1-mediated MAPK pathway. PC-12 cells were split up into Control, DMSO, CORT, as well as CORT + AsVI. The CORT model cohort was transfected with pcDNA3.1-NRG-1, and the CORT + AsVI cohort was transfected with shNRG-1. (**A**) The cell viability was established by the CCK-8 assay. (**B**) HPLC analysis was conducted to measure the DA release levels for various therapies in PC12 cells. (**C**) HPLC analysis was conducted to ascertain 5-HT release levels for various therapies in PC12 cells. (**D**) A quantitative real-time PCR method was carried out to identify the expression level of NRG-1 mRNA in different treated PC12 cells. (**E**,**F**) Western blot for NRG-1, MEK1, p-MEK1, ERK1/2, as well as the p-ERK1/2 protein for different treatments in PC12 cells. All the experiments were conducted thrice. Data are articulated as Mean ± SD. Distinct letters (a, b, c, d) denote significant differences in various cohorts. *p* < 0.05.

To determine whether the NRG-1 signaling is obligatory for the protection of AsVI against major depression, we transfected the PC12 cells with pcDNA3.1-NRG-1 in the CORT model cohort to overexpress NRG-1 while transfecting the cells with shNRG-1 in the CORT + AsVI cohort to inhibit the NRG-1 expression. CCK-8 assay indicated that the overexpression of NRG-1 significantly increased the cell viability in the CORT-induced cellular model of major depression, indicating the neuroprotective role of NRG-1 in major depression. Furthermore, NRG-1 knockdown with ShNRG-1 markedly abolished the protection of AsVI on PC-12 cells (Figure 7A). Consistently, NRG-1 overexpression raised the levels of DA expression (Figure 7B) and 5-HT (Figure 7C), while NRG-1 knockdown decreased 5-HT and DA levels in the CORT-induced cellular model of major depression. As expected, the expression of NRG-1, p-MEK1 and p-ERK1/2 functions were elevated in the NRG-1 overexpressing CORT cohort but reduced in the NRG-1 silencing CORT + AsVI cohort (Figure 7D–F). These findings further suggest that the upregulation of the NRG-1-mediated MAPK pathway could be essential for the protection of AsVI against the CORT-induced cellular model of major depression.

## 3. Discussion

To our knowledge, this is the first study to employ AsVI for the treatment of PSD. PSD is the most frequent neuropsychiatric consequence that occurs in approximately one-third of stroke survivors and affects the quality of life, rehabilitation outcome, functional outcome, and fatality rate. In order to evaluate the pharmacological antidepressant activities of AsVI on PSD, we adopted the rat PSD models in vivo by using MCAO plus CUMS, which showed significant depressive behaviors such as body weight loss, decreased sucrose preference and lower motivation. Treatment with AsVI ameliorated the decreased body weight, sucrose preference and motivation in rat PSD models in vivo. Typical behavioral tests, including SPT, FST, OFT, and Rotarod test, showed that AsVI treatment significantly reduced depression-like behaviors and reversed the impaired physical function in PSD rats. These findings indicate that AsVI could be a promising treatment for PSD.

Depression is closely associated with decreasing levels of brain neurotransmitters, including DA, NE, and 5-HT activity [39,40,41]. During PSD, the axons containing these neurotransmitters between the cerebral cortex and brainstem may be compromised, resulting in an imbalance in DA, NE, and 5-HT synthesis across the brain [42]. Almost all antidepressant approaches have been demonstrated to improve the transmission of neurotransmitters in the brain of laboratory animals [43]. Here, we found that AsVI upregulated 5-HT and DA levels in the PSD rat’s brain tissues in vivo. Besides, AsVI treatment consistently stimulated increased levels of DA/5-HT release in PC-12 cells, associated with a reduction of apoptosis in a CORT-triggered major depression model in vitro. These data indicated that AsVI could be an antidepressant to enhance neurotransmitter levels and protect cells against apoptosis.

Hippocampal neurogenesis has been found to be strongly linked to mood and cognitive function after a stroke [44]. Cumulative research findings have illustrated that stress suppresses neurogenesis in the hippocampus dentate gyrus (DG) [45]. The existing evidence corroborates that PSD is linked to decreased neurogenesis after ischemic stroke [41]. The antidepressant treatment ameliorates depression-like behavior and promotes adult hippocampal neurogenesis. Our previous work demonstrated that AsVI promoted the proliferation of neural stem cells in vitro and increased the neurogenesis in the transient cerebral ischemic hippocampus in vivo. AsVI treatment improved the impaired both physical and neurological cognitive functions in post-ischemic stroke rats by effectively activating EGFR/MAPK signaling cascades [38]. As far as we know, this is the first experimental evidence illustrating the therapeutic possibility of AsVI against PSD by enhancing post-stroke neurogenesis.

Neurotrophic factors have been shown to be crucial in the mediation of behavioral reactions to antidepressants [46]. NRG-1 is widely recognized as a growth factor that has a prospective neuroprotective ability in rat models of permanent focal cerebral ischemia [47]. The ERK, which is affiliated with the mitogen-activated protein kinase (MAPK) family, has been shown to be vital for cell growth, differentiation, as well as survival [48]. In a focal cerebral ischemia model in rats, MAPK/ERK1/2 signaling pathway is known to be crucial in the mediation of neuronal cell survival against apoptosis [49]. Moreover, the triggering of MEK1/2 and ERK1/2 was involved in the neuroprotective impacts of brain-derived neurotrophic factor (BDNF) against the death of apoptotic cells in a transient focal cerebral ischemia rat model [50,51]. NRG-1 is an upstream modulator of the phosphorylation of ERK signaling cascade and stimulates hippocampal neurite extension and arborization [52]. Here, we demonstrated that AsVI increased the expression of NRG-1 to activate the MEK1/ERK1/2 signaling pathway. Overexpression of NRG-1 could reduce the PC12 neuronal cell injury, while knockdown of NRG-1 abolished the protection of AsVI on the CORT-induced cellular model of major depression. The MEK1/ERK1/2 activation showed a similar pattern corresponding to the changes of NRG-1. Such results strongly suggest that the NRG-1/MEK-1-ERK1/2 pathway is essential for the protective effects of AsVI on the post-stroke depression model.

## 4. Materials and Methods

### 4.1. Animal Cohort and Model Establishment

An aggregate of 60 male Sprague Dawley (SD) rats whose weight ranged between 200–240 g was procured from Charles River (Beijing, China) and housed in plastic enclosures at ambient temperature (22 ± 2 °C) with a relative humidity between 50–60% and unrestricted access to basic rat die and tap water at the experimental center of Traditional Chinese Medicine Hospital of Guangdong Province. According to the experimental requirement, these rats were categorized randomly into five cohorts (*n* = 12 per cohort): (1) Sham, (2) MCAO, (3) PSD, (4) PSD + NS, and (5) PSD + AsVI cohorts. The intraluminal suture method was utilized to induce MCAO, as formerly explained [53]. Succinctly, 4% isoflurane with 64% N_2_O and 30% O_2_ was utilized to anesthetize the rats. A midline cervical incision was made to expose the left-sided carotid arteries. This was followed by distally dissecting and isolating the left external carotid artery via the coagulation of its branches and placement of a distal ligation before the operation. A piece of 3–0 sterile monofilament nylon suture, whose tip was heated gently to make it round, was inserted into the left anterior cerebral artery through the lumen of the left external carotid artery stump and the left internal carotid artery to occlude the left middle cerebral artery at its origin. A sudden reduction in regional cerebral perfusion to below 30 percent of the baseline value was considered to achieve focal ischemia. Body temperature was controlled at 36.5–37 °C during the surgery. The intraluminal suture was removed after 90 min of cerebral ischemia to allow reperfusion to occur. The sham control cohort underwent the same surgical process without suture occlusion. The PSD model was established according to a recent report: Separately confined rats in MCAO cohorts were exposed to CUMS with seven distinct stimuli (ice-water swimming, electric shock to foot, tail clamping, behavioral restriction, fasting, wet litter, and water deprivation) [54,55].

As described in previous literature [56], CUMS began 7 days after the surgical operation and continued for 5 weeks. The rats in cohorts 5 and 4 were intravenously injected with AsVI (2 μg/kg wt., dissolved in saline, Shanghai Institute of Meteria Medica with a purity of 99%) or a similar amount of normal saline (NS) as vehicle control at 2 h following MCAO ischemia one time every day for 7 days. Sterile/aseptic methods were employed when performing all animal surgical procedures with approval from the Institutional Animal Care and Use Committee at the hospital before the commencement of the experiment (Approve No. BYL20190202). After the establishment of the animal model, the body weight of the rats was regularly recorded every five days for one month. At 42 days following MCAO ischemia-reperfusion damage, the rats were killed. An important concept in stroke research is the distinction between the “core” and the “penumbra” of the infarct, and many studies have indicated that neuron death in the penumbra could be prevented with neuroprotective drugs, so we chose the penumbra area striatum and hippocampus for our study [57].

Half of the rats’ brain tissues were removed, followed by fixing in 4% paraformaldehyde (PFA) at a temperature of 4 °C over the night and dipped in 30% sucrose for an additional 24 h and subsequently placed in storage at a temperature of 4 °C. The other half of the brain tissues were collected and frozen in liquid nitrogen for 24 h and then put in storage at −80 °C.

### 4.2. Behavioral Tests

#### 4.2.1. Sucrose Preference Test (SPT)

As mentioned previously [58], Reward behavior in rats was assessed via the sucrose preference test (SPT). Before the SPT, the rats in each cohort were deprived of water and food for a period of 24 h. Subsequently, rats were provided with 2 bottles in the home cage for 60 min, with 1 comprising standardized drinking water while the other comprised of 1% sucrose solution. The 2 bottles were weighed after 1 h, and the consumption of sucrose solution and water were analyzed utilizing the algorithm: SPT (%) = Sucrose solution intake/total intake × 100%. During the process of developing the model and implementing the therapy, the sucrose preference was assessed after every 5 days.

#### 4.2.2. Open Field Test (OFT)

The OFT tests were used to evaluate the despair of rats [59]. Tests were conducted every five days on the rats from different cohorts using an open box (100 cm × 100 cm × 50 cm in size) with black walls and an automated data collecting and processing program (Shanghai Xin-ruan Information Technology Co., Shanghai, China). In this box, rats were kept in an open field response tank for five minutes, and their horizontal and vertical motions were captured utilizing a digital camera. The distance of spontaneous moves (SM), the percentage of duration time spent in the center square (duration = time spent in the center square (s)/total time (s) × 100%), and the speed (movement distance/the corresponding duration time) were automatically analyzed.

#### 4.2.3. Forced Swim Test (FST)

FST was applied to assess depression-like behaviors [60]. Training swimming was performed for 15 min, and all the rats were returned to the cages 24 h before the FST experiments. On the second day, the rats were permitted to swim for 6 min in a container (60 × 30 × 45 cm) full of water, the maximum height of which is roughly 40 cm (25 ± 1 °C). When a rat stayed passively floating in the water with its snout above the water’s surface, the last 4 min was recorded, and it was deemed immobile. The time spent floating was later scored every 4 days by an investigator blinded to the design.

#### 4.2.4. Rotarod Test (RT)

An accelerating Rotarod test (UgoBasile, Varese, Italy) was performed to evaluate the motor deficiency functions, especially the asymmetry in the forelimb of the rats [61]. The rats were placed on a rotarod cylinder, which was spun at a pace of 4–40 rpm within 5 min. When the rats fell off or hung on the rung, the experiment was ended. The time, rotating speed and distance from the start to the end of the trial were measured, based on which the average speed value was determined.

### 4.3. Cell Culture and Cohort

As mentioned previously [62,63], corticosterone (CORT) is widely recognized for inducing apoptosis in pheochromocytoma cells (PC12) and has frequently been used in major depression models [62,63]. Medications that could mitigate neurotoxicity induced by CORT are considered to have promising therapeutic potential to treat major depression [43,64]. The PC12 cells were grown in Dulbecco’s Modified Eagle’s Medium comprising 5 percent fetal bovine serum (FBS, Biosharp, BL1407A, Hefei, China) and 10 percent of horse serum(Biosharp, BL209A, Hefei, China), streptomycin (100 μg/mL), and penicillin (100 unit/mL; Biosharp, BL505A, Hefei, China) which were sustained in a moistened incubator with 5 percent CO_2_ concentration at a temperature of 37 °C. The PC12 cells were incubated with 300 μM CORT(Sigma-Aldrich, 802905, St. Louis, MA, USA; dissolved in DMSO) for 24 h to construct the major depression cellular model. To test the impact of AsVI on the major depression cellular model, we designed cohorts, including non-treated Control, DMSO, CORT, and CORT + AsVI. AsVI was dissolved in 0.5 percent DMSO, and the ending concentration of 100 nM was introduced into the culture medium before CORT treatment. To investigate the antidepressant effects of NRG-1, we transfected the PC12 cells in the CORT model cohort with pcDNA3.1-NRG-1 to overexpress NRG-1 while transfected the cells in CORT + AsVI cohort with shNRG-1 to inhibit the NRG-1 expression. NRG-1 overexpression plasmids were obtained from the Vipotion (Guangzhou, China), and the NRG-1 knockdown plasmids shNRG-1 were purchased from GenePharma (Shanghai, China). The transfection efficiency exceeded 85%.

### 4.4. HPLC Analysis of DA/5-HT Level Release

HPLC analysis was conducted to measure the neurotransmitter in brain regions or PC12 cells. In brief, the hippocampus and striatum were obtained and homogenized. Meanwhile, the culture media of PC12 cells were collected after various treatments, including Control, DMSO, CORT, CORT + NC, CORT + Nrg-1, CORT + AsVI, CORT + shNrg-1 + AsVI for 24 h. For DA, the mobile phase consisted of methanol (Sigma-Aldrich, 34885, St. Louis, MA, USA) /0.01 M KH2PO4 (Sigma-Aldrich, RDD037, St. Louis, MA, USA; 10:90,*v*/*v*, pH 3.5) at a flow rate of 0.5 mL/min and was detected at a wavelength of 280 nm. With regards to 5-HT, the isolation was performed using a mobile phase comprising of methanol/CH3COONa (Sigma-Aldrich, S2889, St. Louis, MA, USA; 60:40, *v*/*v*, 0.05 M) with a flow rate of 0.5 mL/min, and detected a wavelength of 275 nm. Standard DA/5-HT samples were used to obtain a standard curve, and the concentrations of DA/5-HT in the brain regions or media were determined from the standard curve.

### 4.5. Immunostaining

For the in vivo immunostaining analysis, brain tissues (including hippocampus and striatum), harvested at 42 days following MCAO ischemia-reperfusion damage, were fixed in 4% paraformaldehyde (Biosharp, BL539A, Hefei, China), embedded in paraffin wax, and then cut into 4 μm parts utilizing a vibratome (Leica). Incubation with the main antibody against NRG-1 (Abcam, ab180808, 1:50, Waltham, MA, USA) was performed utilizing super fluorescein isothiocyanate immunofluorescence detection system kit (Bioworld, Bloomington, MN, USA) as per the protocol stipulated by the manufacturer. The nucleus was labeled using 4’,6-diamidino-2-phenylindole (DAPI, Biosharp, BL120A, Hefei, China). Eventually, a fluorescence microscope (Leica, Wetzlar, Germany) was utilized to examine the specimens.

### 4.6. Western Blot

Total proteins were isolated from the stored brain tissues containing hippocampus, striatum, and cortex or PC12 cells using a protein extraction kit (KenGen, Biotech, Co., Ltd., Nanjing, China) with a 1% protease inhibitor cocktail (Biosharp, BL630B, Hefei, China), and protein concentration was ascertained by bicinchoninic acid assay (KenGen, Biotech, Co., Ltd., Nanjing, China) as per the instructions stipulated by the manufacturer. Equivalent quantities of protein (30 μg) were put onto a polyvinylidenedifluoride (PVDF, Bio-Rad, California, CA, USA) membrane and isolated utilizing sodium dodecyl sulfate (SDS) polyacrylamide gel. Subsequently, blocking of the membrane was done using 5% (*w*/*v*) bovine serum albumin (BSA) in Tris Buffered Saline with Tween 20 (TBST) for a duration of 1 h followed by incubation with primary antibodies against NRG-1 (Abcam, ab180808, 1:1000), MEK1 (Abcam, ab32091, 1:2000), p-MEK1 (phosphor S298, Abcam, ab96379, 1:1500), ERK1/2 (Abcam, ab17942, 1:2000), p-ERK1/2 (Phospho-p44/42, Cell Signaling Technology, #9101S, 1:1000) and GAPDH (BOSTER, BM1985, 1:500) over the night at a temperature of 4 °C. Following three washes using TBST, incubation of the membrane was done using horseradish peroxidase-conjugated secondary antibody (goat anti-rabbit IgG, Abcam, ab6721, 1:10,000 or goat anti-mouse IgG, Abcam, ab6728, 1:5000) for 1.5 h at ambient temperature. Finally, bands were observed utilizing immuno-western star ECL detection kits (Thermo Fisher Scientific, Inc.). The film was scanned using a bio-imaging analyzer (Bio-Rad, USA), and Densitometric quantification was conducted utilizing Bio-Rad Quantity One software v.4.6.3 (Bio-Rad Laboratories, Hercules, CA, USA).

### 4.7. Cell Viability Assay

Assessment of cell viability was done utilizing the Cell Counting Kit-8 (CCK-8, Dojindo Molecular Technologies, Kumamoto, Japan). In brief, 100 μL of PC12 cells, Nrg-1 transfected PC12 cells, and shNrg-1 transfected PC12 cells (1 × 105/mL) underwent seeding in a 96-well plate for 12 h and subsequently treated using 0.5 percent DMSO, 300 μM CORT and/or 100 nM AsVI for 24 h. Afterward, the removal of the medium was done, and each of the wells was grown with 10 μL of CCK-8 solution and 90 μL of culture medium at a temperature of 37 °C for 2 h. Finally, the optical density at 450 nm was assessed using a microplate reader (ELX 800 UV, BIO-TEK, USA).

### 4.8. Statistical Analysis

The SPSS software (version 18.0, Armonk, NY, USA) was utilized to perform all statistical analyses. All data are indicated as means ± standard deviation (SD). Statistical multiple comparisons were carried out utilizing a one-way analysis of variance (ANOVA) with Tukey’s post hoc test. *p* values below 0.05 were judged significant.

## 5. Conclusions

In conclusion, for the first time, we report that AsVI can alleviate depressive behavior, increase neurotransmitter DA/5-HT in brains, and exerts a positive effect on inhibiting neuron apoptosis to protect PSD in vivo and in vitro. The beneficial effects of AsVI treatment could be associated with upregulating NRG-1 mediated the triggering of the MEK1/ERK1/2 signaling pathway.

## Data Availability

Data is contained within the article.
